# Supplementary material for: Polymorphisms in Pfkelch13 domains before and after the introduction of artemisinin-based combination therapy in Southwest Nigeria
Source: PLoS One. 2025 Mar 31;20(3):e0316479. doi: 10.1371/journal.pone.0316479 (PMC11957316; doi:10.1371/journal.pone.0316479)

Samples: 13826
Bases: 1092
Average spacing: 13

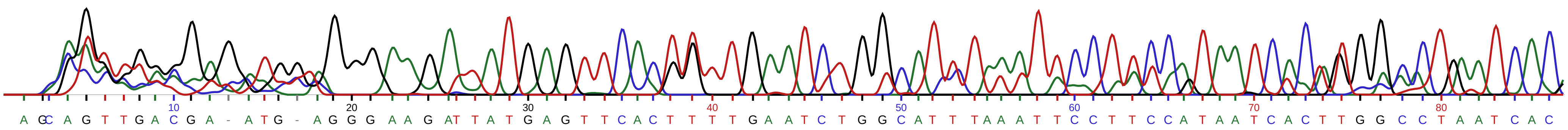

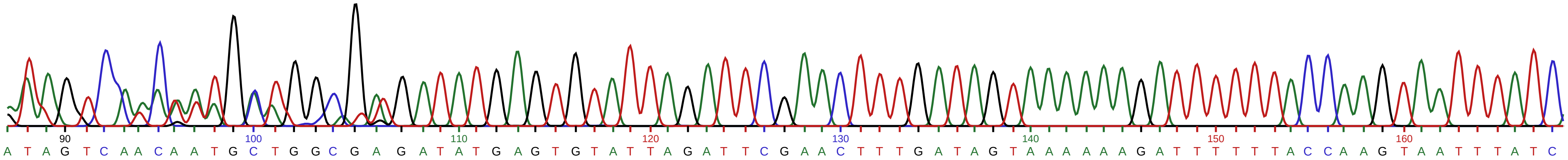

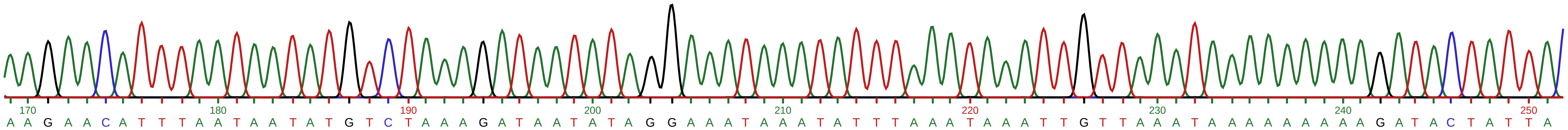

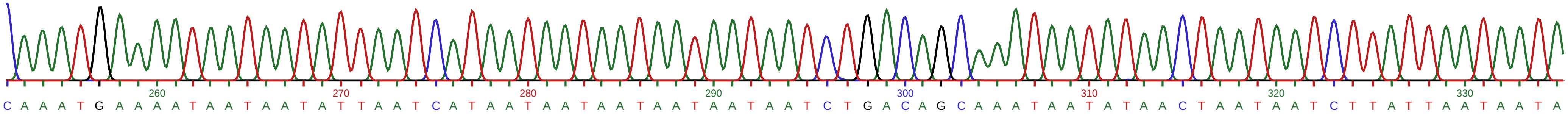

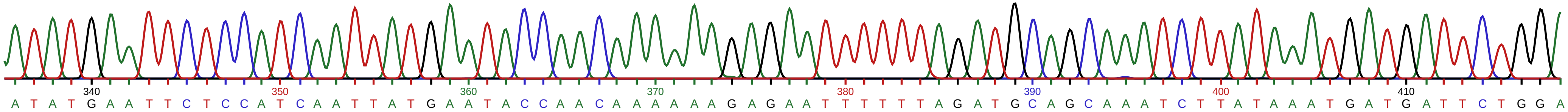

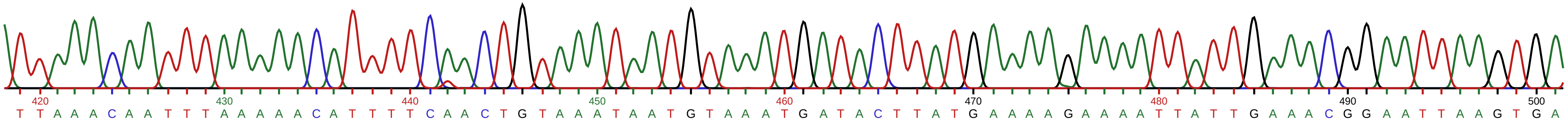

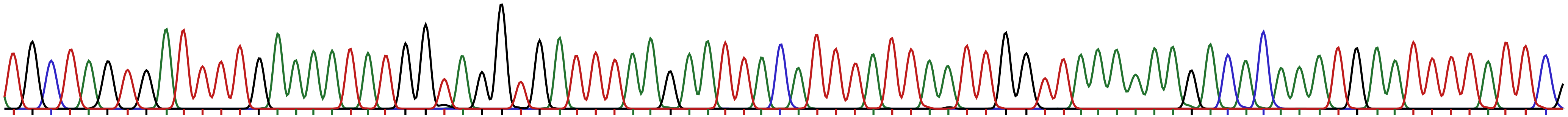

510 520 530 540 550 560 570 580

T G C T A G T G A T T T T G A A A A T A T G G T A G G T G A T T T A A G A A T T A C A T T T A T T T A A T T G G T T A A A A A A G A C A C A A A T G A A T T T T A T T C

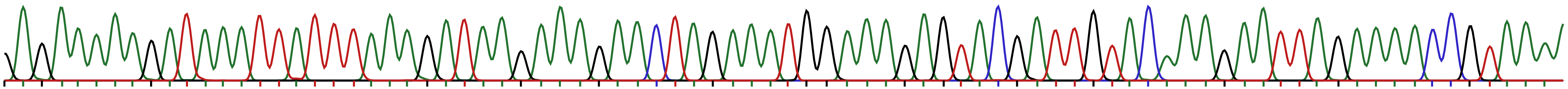

590 600 610 620 630 640 650 660

G A G A A A A A G A T A A A T T A T T T A A A G A T A A G A A A G A A C T A G A A A T G G A A A G A G T A C G A T T G T A C A A A G A A T T A G A A A A C C G T A A A

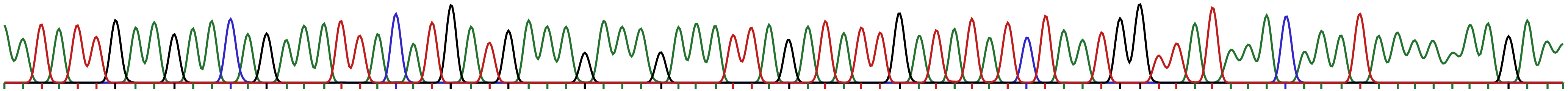

670 680 690 700 710 720 730 740 750

A A T A T T G A A G A A C A G A A A T T A C A T G A T G A A A G A A A G A A A T T A G A T A T T G A T A T A T C T A A T G G T T A T A A A C A A A T A A A A A A A G A A A

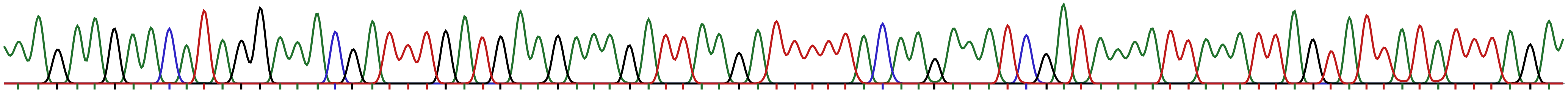

760 770 780 790 800 810 820 830

A A G A A G A A C A T A G G A A A C G A T T T G A T G A A G A A A G A T T A A G A T T T T T A C A A G A A A T C G A T A A A A T T A A A T T A G T A T T A T A T T T A G A

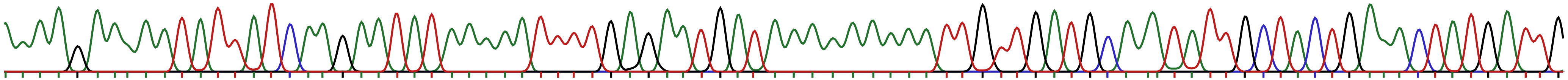

840 850 860 870 880 890 900 910 920

A A A A G A AA A A T A T T A T C AA G A A T A T A A A A A T T T T G A G A A T G A T A A A A A A A A A A T T G T T G A T G C A AA T A T T G C T A C T G A A A C T A T G A T T G

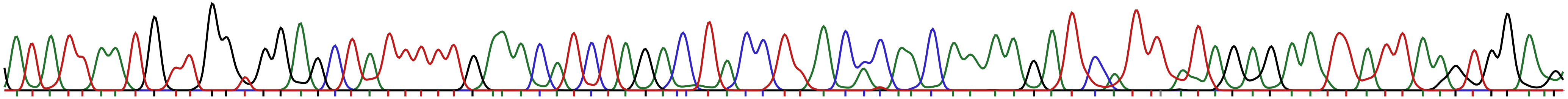

930 940 950 960 970 980 990 1000 1010

A T A T T A A T G T T G G T G G A G C T A T T T T T G AA A C A T C T A G A CC T A C C T T A C C C AA C A A A A G A T C A T T - A T A G A G A A T T A T T A A G T G G A AG

Samples: 13826
Bases: 1092
Average spacing: 13

8/17/2022

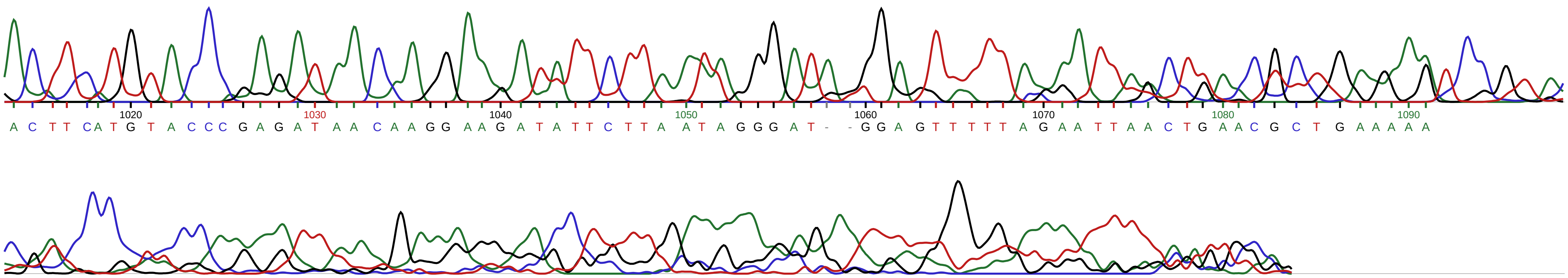

Supplement: Supporting information 2 — (ZIP) [file pone.0316479.s002.zip › 038KN2F_PREMIX_Plate_KELCH1_H05.pdf]
